# Single-view geometric calibration for C-arm inverse geometry CT

**DOI:** 10.1117/1.JMI.4.1.013506

**Published:** 2017-03-20

**Authors:** Jordan M. Slagowski, David A. P. Dunkerley, Charles R. Hatt, Michael A. Speidel

**Affiliations:** a University of Wisconsin, Department of Medical Physics, Madison, Wisconsin, United States; b University of Wisconsin, Department of Biomedical Engineering, Madison, Wisconsin, United States; c University of Wisconsin, Department of Medicine, Madison, Wisconsin, United States

**Keywords:** inverse geometry, scanning-beam digital x-ray, C-arm calibration, computed tomography

## Abstract

Accurate and artifact-free reconstruction of tomographic images requires precise knowledge of the imaging system geometry. A projection matrix-based calibration method to enable C-arm inverse geometry CT (IGCT) is proposed. The method is evaluated for scanning-beam digital x-ray (SBDX), a C-arm mounted inverse geometry fluoroscopic technology. A helical configuration of fiducials is imaged at each gantry angle in a rotational acquisition. For each gantry angle, digital tomosynthesis is performed at multiple planes and a composite image analogous to a cone-beam projection is generated from the plane stack. The geometry of the C-arm, source array, and detector array is determined at each angle by constructing a parameterized three-dimensional-to-two-dimensional projection matrix that minimizes the sum-of-squared deviations between measured and projected fiducial coordinates. Simulations were used to evaluate calibration performance with translations and rotations of the source and detector. The relative root-mean-square error in a reconstruction of a numerical thorax phantom was 0.4% using the calibration method versus 7.7% without calibration. In phantom studies, reconstruction of SBDX projections using the proposed method eliminated artifacts present in noncalibrated reconstructions. The proposed IGCT calibration method reduces image artifacts when uncertainties exist in system geometry.

## 1 Introduction

**Fig. 1:**
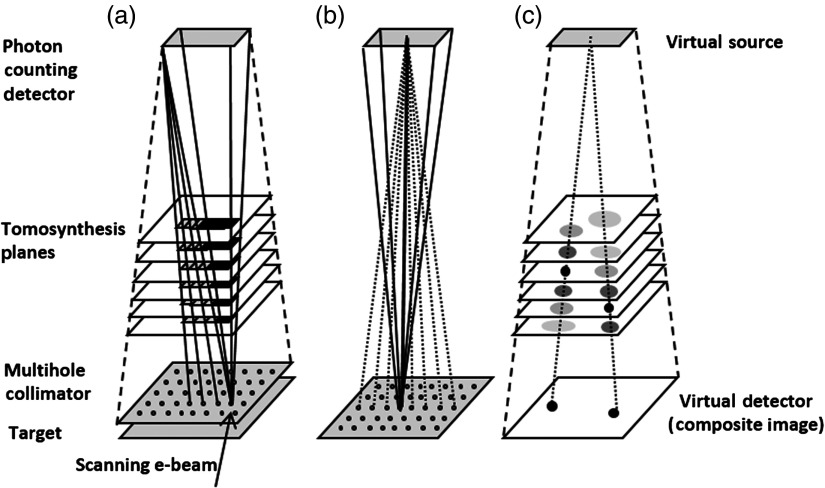
(a) SBDX performs digital tomosynthesis, (b) detector images are backprojected along the ray connecting each source position to the center of the detector, and (c) the composite image is analogous to a 2-D virtual projection image.

Scanning-beam digital x-ray (SBDX) is a low-dose inverse geometry fluoroscopic technology designed for cardiac interventions [Fig. 1(a)].^1^^,^^2^ The SBDX x-ray source consists of a raster scanned electron beam, large-area transmission style target, and multihole collimator. In each fluoroscopic frame, the electron beam visits a two-dimensional (2-D) array of discrete focal spot positions (Fig. 1). X-rays arising from each focal spot are collimated to a small-area detector that captures images as the scan proceeds. The detector images are then reconstructed into full field-of-view images in real time. The SBDX geometry is designed to achieve dose reduction in fluoroscopic applications through a reduction in detected scatter and an increase in entrance field area.^3^ Additionally, since each point in space is imaged from multiple view angles in a frame period, SBDX provides a real-time tomosynthesis capability.^2^ SBDX tomosynthesis has been exploited for a number of applications including frame-by-frame three-dimensional (3-D) tracking of high-contrast objects, such as cardiac catheters,^4^ calibration-free vessel measurements for device sizing,^5^ and stereoscopic fluoroscopy.^6^

Recently, the feasibility of CT-based 3-D cardiac chamber mapping with SBDX was demonstrated through numerical simulations.^7^^,^^8^ An SBDX-derived cardiac chamber surface map could potentially be displayed along with the real-time SBDX 3-D catheter tracking results to facilitate device navigation during an interventional procedure, such as radiofrequency catheter ablation for atrial fibrillation. In practical C-arm-based inverse geometry CT (IGCT), uncertainties in the imaging geometry may exist due to manufacturing tolerances, nonideal source and detector alignment, or C-arm deflection during rotation. An incorrect mapping between the assumed 3-D object coordinate system and the projection acquisition system has been shown to degrade spatial resolution and can introduce image artifacts.^9^

A proposed IGCT calibration method by Schmidt et al.^10^ estimated four parameters describing the location and orientation of a common axis-of-rotation for the source and detector arrays of a table-top IGCT system with a rotating stage. A second approach proposed by Baek et al.^11^ improved on the four parameter method by estimating individual source coordinates for an 8 spot x-ray source array mounted on a rotating gantry. Baek’s approach also determined four parameters describing the axis-of-rotation. For C-arm CT, it is desirable that geometric calibration be performed independently at each view angle. Uncertainties in the C-arm geometry may be larger due to the use of a nonrigid C-arm compared to more stable gantry-based systems. This work proposes a gantry calibration method for IGCT that is motivated by the projection matrix (P-matrix) technique often used in cone-beam CT.^12^ The proposed method performs a calibration for each gantry position independent of the other acquired view angles.

The ability of the proposed method to recover parameters describing SBDX system geometry is examined using numerical simulations. Finally, experimental SBDX data acquired with a rotating phantom stage are used to demonstrate that the proposed method reduces image artifacts caused by geometric uncertainty.

## 2 Methods

### 2.1 Scanning-Beam Digital X-ray Scanning Principles

The SBDX system geometry studied in this paper is summarized in Table 1. SBDX uses an electromagnetically scanned electron beam incident upon a large-area transmission style tungsten target. For the cardiac imaging mode considered in this work, the electron beam is raster scanned over a 71×71 subset of the available 100×100 source focal spot positions every 1/15  s. A multihole collimator positioned beyond the target defines a series of narrow overlapping x-ray beams convergent upon a 10.6  cm×5.3  cm photon-counting detector array with 2-mm-thick CdTe. The nominal source–detector distance (SDD) is 1500 mm and the nominal source-to-isocenter distance is 450 mm. The geometric relationship among the narrow beam projections is constrained by the precise and rigid geometry of the SBDX collimator and the fixed detector position.

**Table 1:** SBDX system geometry.

SDD	1500 mm
SAD	450 mm
Focal spot positions	71×71
Focal spot pitch	2.3×2.3 mm
Native detector array	320×160
Native detector element pitch	0.33 mm
Detector bin mode	2×2

In the 71×71, 15  frame/s scanning mode, each focal spot position is visited by the electron beam 8 times per frame. The raster scanning proceeds blockwise, with all eight passes performed on a block of three rows before proceeding on to the next block. The electron beam dwell time at a focal spot position is 1.04  μs and the travel time between positions is 0.24  μs.^2^ The detector captures an image every 1.28  μs. The time difference between the first and last x-ray beam illuminations of a fixed point in the field-of-view is referred to as the effective pulse width. For a point at isocenter, the effective pulse width is 8.9 ms.^2^

### 2.2 Scanning-Beam Digital X-ray Image Reconstruction

SBDX has a tomosynthesis imaging capability due to the use of inverse geometry beam scanning. A live display analogous to conventional 15 fps fluoroscopy is generated using a graphics processing unit-based real-time image reconstructor.^2^ Each displayed 2-D image frame is generated through a two stage reconstruction procedure. First, shift-and-add (SAA) digital tomosynthesis is performed to generate a stack of 32 single plane images with 5-mm plane spacing [Fig. 1(a)]. Each detector image acquired in a frame period is backprojected and summed at the stack of reconstruction planes.^1^ The pixel width in each reconstructed plane is defined by dividing the shift distance between adjacent backprojected detector images into 10 pixels. Each detector element value is divided among the pixels it overlaps according to the area of overlap. By this convention for defining pixels, each single-plane image has a fixed number of pixels (710×710) and the physical pixel dimension increases as the distance from detector to reconstruction plane increases. The isocenter-plane pixel width is 0.161 mm. As shown in Fig. 1(b), the pixel centers for the stack of tomosynthesis images are defined such that a fixed pixel position (e.g., row 100 and column 100) in the stack corresponds to a ray originating at the detector center.

After digital tomosynthesis, a gradient filtering procedure is applied to each of the single-plane images to identify local regions of high sharpness and contrast. The final 2-D “composite” image is then formed by selecting, for each pixel position, the pixel value from the single-plane image with highest contrast and sharpness. Due to the geometry of the tomosynthesis pixel centers and the compositing procedure, the final composite image can be viewed as an inverted “virtual” cone-beam projection of the in-focus objects in the patient volume [see Fig. 1(c)]. A virtual SBDX projection originates at the center of the detector and falls on the source plane. Noting that the lateral distance between rays drawn from the detector center to adjacent focal spot positions is always divided into 10 pixels during tomosynthesis reconstruction, the pitch of the virtual detector elements at the source plane is equal to the focal spot pitch (2.3 mm) divided by 10 pixels or 0.23 mm.

### 2.3 Geometric Calibration

Since the SBDX composite image can be viewed as a virtual cone-beam projection, a projection matrix approach can be used to estimate the geometric parameters of the SBDX system for calibration. The 3×4 projection matrix P maps a point (x, y, z) in the 3-D object frame-of-reference to homogeneous coordinates (λu, λv, λ)

$${\left[\lambda u,\lambda v,\lambda \right]}^{T}=P{\left[x,y,z,1\right]}^{T}.$$ (1)

The parameter λ is a scaling factor. The detector indices (u,v) corresponding to the projection of point (x,y,z) onto the virtual detector plane may be obtained by dividing the homogenous coordinates by λ.

The unknown projection matrix P is written as a product of three matrices P=KRT. Here, K describes the virtual projection geometry’s intrinsic parameters, R is a rotation matrix, and T is a translation matrix. K depends on SDD, pitch between virtual detector elements ($s_{p}$), and the coordinates $\left(u_{o},v_{o}\right)$, which define the piercing point on the virtual detector. R depends on three angles $\left(\theta_{x},\theta_{y},\theta_{z}\right)$ describing the rotations about the three principal axes. T depends on the location of the virtual source point $\left(x_{s},y_{s},z_{s}\right)$ in the 3-D object coordinate system. Denoting $c_{j}=\cos(\theta_{j})$ and $s_{j}=\sin(\theta_{j})$, the projection matrix P is given by

$$P=\left[\begin{array}{ccc} -u_{o} & \mathrm{SDD}/s_{p} & 0\\ -v_{o} & 0 & \mathrm{SDD}/s_{p}\\ -1 & 0 & 0 \end{array}\right][\begin{array}{ccc} 1 & 0 & 0\\ 0 & c_{x} & s_{x}\\ 0 & -s_{x} & c_{x} \end{array}]\times [\begin{array}{ccc} c_{y} & 0 & -s_{y}\\ 0 & 1 & 0\\ s_{y} & 0 & c_{y} \end{array}][\begin{array}{ccc} c_{z} & s_{z} & 0\\ -s_{z} & c_{z} & 0\\ 0 & 0 & 1 \end{array}]\left[\begin{array}{cccc} 1 & 0 & 0 & -x_{s}\\ 0 & 1 & 0 & -y_{s}\\ 0 & 0 & 1 & -z_{s} \end{array}\right].$$ (2)

**Fig. 2:**
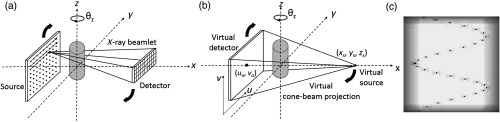
(a) SBDX CT acquisition consisting of source scanning and gantry rotation about the z-axis, (b) matching virtual cone-beam projection geometry, and (c) example composite image (virtual projection) of the helix phantom.

The P matrix is parameterized by a vector, ξ, consisting of nine elements, $\xi =[\mathrm{SDD},u_{o},v_{o},\theta_{x},\theta_{y},\theta_{z},x_{s},y_{s},z_{s}]$. The pitch between virtual detector elements, $s_{p}$, equals 0.23 mm. A calibration phantom containing a known helical configuration of N high-contrast point-like markers is then imaged [Fig. 2(a)]. The virtual detector coordinates $\left(u_{i},v_{i}\right)$ of the projections of the markers are then determined using a center-of-mass technique. The geometric parameters describing the IGCT system are estimated by minimizing the sum-of-squared differences between the measured positions of the markers $\left(u_{i},v_{i}\right)$ and the P-matrix-projected marker detector coordinates [$u_{i}(\xi ),v_{i}(\xi )$]

ξ^=arg minξ1N∑i=1N{[ui(ξ)−ui]2+  [vi(ξ)−vi]2}. (3)

The optimization was performed using a quasi-Newton method, with initial parameters set to the nominal system geometry described in Table 1.

### 2.4 Simulations

The performance of the proposed geometric calibration method was evaluated through numerical simulations of SBDX CT data acquisition with simulated deviations from the nominal SBDX geometry. A noise-free SBDX CT data acquisition consisting of 210 view angles uniformly distributed over 210 deg was simulated for a known helical configuration of 30 spherical steel fiducials. The helix phantom was centered at isocenter with its long axis aligned with the axis-of-rotation [z-axis; see Fig. 2(a)]. The SBDX source and detector were translated 1 mm in the +y–direction, and the source and detector arrays were rotated 1 deg about the x-axis to mimic a hypothetical system misalignment. For each gantry orientation, a composite image [Fig. 2(c)] was reconstructed and a 3×4 projection matrix P was derived.

The CT data acquisition scheme was then repeated for a numerical thorax phantom. Reconstruction was performed using a gridded filtered back-projection (gFBP) algorithm^13^ with and without geometric calibration to assess the proposed method’s ability to reduce image artifacts. To quantify the accuracy of the reconstruction with and without calibration, the relative root-mean-squared-error (rRMSE) was calculated versus the known ground truth. To isolate and quantify errors related only to geometric calibration, the rRMSE was also computed versus a gFBP reconstruction of the thorax phantom accounting for the known deviations in system geometry. The rRMSE is defined as

$$r\mathrm{RMSE}(x)=\sqrt{\frac{1}{N}\overset{N}{\underset{i}{\sum }}{\left[\frac{x_{i}-x_{i}^{\mathrm{ref}}}{\max(x^{\mathrm{ref}})-\min(x^{\mathrm{ref}})}\right]}^{2}}.$$ (4)

### 2.5 Sensitivity Analysis

The sensitivity of the method to uncertainties in SDD, translations, and rotations was investigated through numerical simulations. The nominal SBDX system geometry parameters defined in Table 1 were perturbed by varying amounts for two scenarios. In the first scenario, the SDD and source–axis-distance (SAD) were varied by 1 mm. The SBDX source and detector were translated 1 mm in the y-direction, 1 mm in the z-direction, and rotated 1 deg about the x-axis, 1 deg about the y-axis, and 1 deg about the z-axis. For the second scenario, the SDD and SAD were varied by 5 mm; the source and detector were translated 5 mm in the y-direction, 5 mm in the z-direction, and rotated 5 deg about each of the x-, y-, and z-axes. For each scenario, the proposed calibration method was used to estimate the system geometry and was compared versus the known perturbations. The mean error and standard deviation in the estimated geometric parameters were determined for 210 gantry view angles evenly distributed over 210 deg.

### 2.6 Experimental Validation

**Fig. 3:**
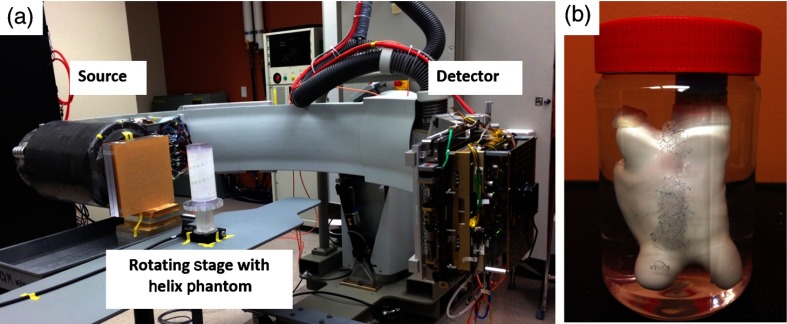
(a) SBDX CT data acquisition was simulated by rotating the C-arm to a horizontal position and rotating the imaging object. The helix calibration object is shown. (b) A custom-made anatomic chamber model placed in an 85-mm-diameter water cylinder.

The proposed calibration method was tested in three phantom studies using projection data acquired with the SBDX system. For each study, a geometric calibration procedure was followed. The SBDX gantry was rotated 90 deg to a lateral angulation and a helix phantom was placed on a rotating stage at isocenter [Fig. 3(a)]. The calibration phantom was constructed out of poly(methyl methacrylate) (PMMA) and consists of a known helical configuration of 41 spherical steel fiducials.^6^ The diameter of the fiducials was 1/16  in. The angular pitch and increment between fiducials along the z-axis were 22.5 deg and 0.15 in., respectively. The phantom contains a 3/32-in.-diameter reference fiducial, which is larger than the other fiducials. The larger fiducial can be used to relate the known 3-D fiducial coordinates to the 2-D projection coordinates using connected component analysis. Only fiducials appearing in the CT field-of-view are used for calibration. The outer diameter of the phantom measured 4 in. and the wall thickness was 3/8  in.

The helix phantom was imaged using a continuous stage rotation with an angular velocity of 2 deg per second over a 220-deg short-scan range. A step-and-shoot technique was used by selecting every 7th frame of the complete projection dataset resulting in 236 view angles with an angular increment of 0.93 deg. Imaging was performed at 100 kV tube potential, 40 mA peak tube current (19% full power), with a 71×71, 15  frames/s scanning technique. Acrylic with a thickness of 7 cm was placed in the beam before the helix phantom. For each view angle, a composite image was reconstructed and a 3×4 projection matrix P was derived.

#### 2.6.1 Atrium phantom

In the first phantom study, a custom-made phantom designed to resemble a left atrium was imaged. This phantom was chosen because the SBDX system is being investigated for the task of 3-D imaging of a cardiac chamber during an interventional procedure. The hollow atrium was filled with 3 ml iohexol contrast agent (Omnipaque 350, GE Healthcare, Waukesha, Wisconsin) diluted with 88 ml of water and placed in an 85-mm-diameter cylinder of deionized water that fits entirely within the SBDX field-of-view [Fig. 3(b)]. Six 1/16-in.-diameter aluminum fiducials were attached to the outside of the water cylinder to demonstrate the proposed method’s ability to mitigate artifacts and resolve small high-contrast point-like objects. SBDX projection data were then acquired using the same imaging technique used for the calibration phantom. The atrium phantom was reconstructed using gFBP with and without geometric calibration. Table 2 presents the rebinning algorithm parameters that were used for reconstruction unless otherwise stated.

**Table 2:** Gridded FBP reconstruction parameters.

Number of view angles over 180 deg	480
Rays per 2-D view (columns×rows)	1120×290
2-D view sampling pitch	0.125 mm×0.5 mm
Radial kernel	1.4 mm×2.0 mm
Angular kernel	2.0 deg × 2.0 deg
Reconstruction grid (number of voxels)	512×512×330
Voxel dimensions	0.27 mm×0.27 mm×0.27 mm

#### 2.6.2 Spatial fidelity metric

In the second study, IGCT reconstruction of the helix calibration phantom was performed with and without geometric calibration. The spatial fidelity of a reconstruction was validated by computing the Euclidean distance between each combination of the steel fiducials. The 30 steel fiducials within the CT image volume were segmented and a center-of-mass calculation was performed to determine the 3-D Cartesian coordinates of each fiducial. The separation distance was calculated for each of the 435 pair combinations. As a reference, the separation distances were also calculated from the known helix geometry. The reference fiducial separation distances ranged from 17.0 to 126.8 mm.

#### 2.6.3 Modulation transfer function

The third study measured the modulation transfer function (MTF) from an image of wire phantom reconstructed with geometric calibration. A hollow cylindrical PMMA phantom containing a 0.154-mm-diameter stainless-steel wire at the center was used to measure the line spread function (LSF). The wire phantom was positioned on the rotating stage with the wire near isocenter and parallel to the rotation axis of the stage. SBDX projection data were then acquired using the same imaging technique used for the helix and atrium phantoms. The projection data were reconstructed using the rebinning parameters listed in Table 2 with a targeted reconstruction FOV. A 512×512×512  pixel reconstruction grid was used with isotropic pixel resolution set to half the diameter of the steel wire, 0.077  mm×0.077  mm×0.077  mm. The MTF was calculated following the method of Kayugawa et al.^14^ The LSF was determined by integrating row by row across the image columns within a 41×41  pixel region of interest centered on the wire. An offset correction was applied to the LSF by subtracting the mean value of the three data points at each edge of the LSF. The LSF was normalized to unit area. The normalized LSF was zero padded and the MTF was computed as the magnitude of the fast Fourier transform of the LSF.

## 3 Results

### 3.1 Simulations

The geometric parameters corresponding to the simulated SBDX CT data acquisition were determined at each view angle. The difference between the extracted value and the true value was computed for each parameter of ξ. Table 3 summarizes the mean error and standard deviation in error versus gantry angle, for each parameter. Over all angles, the maximum error in a rotation parameter ($\theta_{x}$, $\theta_{y}$, $\theta_{z}$) was less than 0.02 deg. The maximum error in the virtual source point ($x_{s}$, $y_{s}$, $z_{s}$) was 0.4 mm, and the maximum error in SDD was −0.13  mm. Errors in the ($u_{o}$, $v_{o}$) coordinates were less than the dimension of a virtual detector element.

**Table 3:** Mean, standard deviation, and maximum errors in estimated geometric parameters for simulated 1 mm translation in $y_{s}$ and a 1-deg rotation in $\theta_{x}$.

	$x_{s}$	$y_{s}$ (mm)	$z_{s}$	$\theta_{x}$	$\theta_{y}$ (deg)	$\theta_{z}$	SDD	$u_{o}$ (mm)	$v_{o}$
Mean error	0.0	0.0	0.1	$2.4\times 10^{-4}$	$-6.0\times 10^{-3}$	$-2.0\times 10^{-3}$	0.0	0.0	0.0
Standard deviation	0.1	0.1	0.1	$4.0\times 10^{-3}$	$6.0\times 10^{-3}$	$6.0\times 10^{-3}$	$4.1\times 10^{-2}$	$2.0\times 10^{-4}$	$2.3\times 10^{-4}$
Max error	0.3	−0.3	0.4	$-1.2\times 10^{-2}$	$-2.0\times 10^{-2}$	$-1.6\times 10^{-2}$	$-1.3\times 10^{-1}$	$-6.4\times 10^{-4}$	$-6.8\times 10^{-4}$

**Fig. 4:**
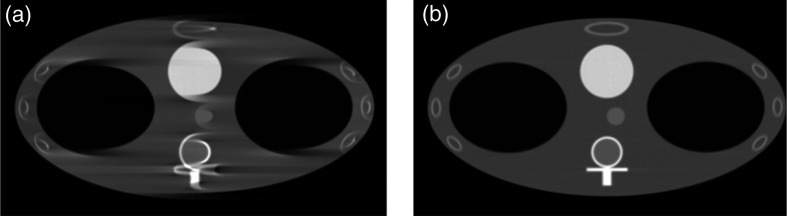
A thorax phantom was simulated with 1 mm translation in $y_{s}$ and a 1-deg rotation in $\theta_{x}$. (a) Is a reconstruction without geometric calibration and (b) is a reconstruction with geometric calibration. Display window is [−800,1000] HU.

A miniature thorax phantom fully enclosed within the SBDX 140 mm field-of-view was used to investigate geometry-calibration-related reconstruction artifacts without the presence of confounding truncation artifacts. The numerical thorax phantom was reconstructed without [Fig. 4(a)] and with [Fig. 4(b)] geometric calibration using gFBP. Figure 4(a) shows significant artifacts and distortion of structures caused by the failure to account for the translation and rotation of the system geometry. These artifacts were removed in the image reconstructed with the proposed calibration technique. The rRMSE was calculated versus the known ground truth to quantify reconstruction accuracy. The rRMSE was 1.3% using the proposed geometric calibration method and 8.0% without geometric calibration. The rRMSE was also calculated versus an image reconstructed using gFBP with exactly known geometry to isolate errors due only to geometric uncertainties. The rRMSE was 0.4% with geometric calibration, versus 7.7% without geometric calibration.

### 3.2 Sensitivity Analysis

Tables 4 and 5 summarize geometry parameter recovery for additional deviations from the nominal geometry. A total of 210 gantry view angles were evaluated for each case. Table 4 presents results where the SDD and SAD were varied by 1 mm, the source and detector arrays were translated +1  mm and +1  mm along the y- and z-axes, and rotated +1  deg about each of the x-, y-, and z-axes. Table 5 presents results where the SDD and SAD were varied by 5 mm, the source and detector arrays were translated +5  mm along the y-axis, +5  mm along the z-axis, and rotated +5  deg about each of the x-, y-, and z-axes.

**Table 4:** Mean and standard deviation of errors in estimated geometric parameters with simulated deviations from the nominal system geometry. The SDD and SAD were varied 1 mm from nominal values. The detector and source arrays were translated 1 mm along the y-axis and 1 mm along the z-axis. The source and detector arrays were rotated 1 deg about the y-axis, 1 deg about the x-axis, and 1 deg about the z-axis.

$x_{s}$	$y_{s}$ (mm)	$z_{s}$	$\theta_{x}$	$\theta_{y}$ (deg)	$\theta_{z}$	SDD	$u_{o}$ (mm)	$v_{o}$
0.1±0.3	−0.2±0.2	0.1±0.1	$0.0\pm 4.2\times 10^{-3}$	$0.0\pm 5.7\times 10^{-3}$	$0.0\pm 6.1\times 10^{-3}$	$-0.6\pm 4.1\times 10^{-2}$	$0.0\pm 2.8\times 10^{-3}$	$0.0\pm 1.2\times 10^{-2}$

**Table 5:** Mean and standard deviation of errors in estimated geometric parameters with simulated deviations from the nominal system geometry. The SDD and SAD were varied 5 mm from nominal values. The detector and source arrays were translated 5 mm along the y-axis and 5 mm along the z-axis. The source and detector arrays were rotated 5 deg about the y-axis, 5 deg about the x-axis, and 5 deg about the z-axis.

$x_{s}$	$y_{s}$ (mm)	$z_{s}$	$\theta_{x}$	$\theta_{y}$ (deg)	$\theta_{z}$	SDD	$u_{o}$ (mm)	$v_{o}$
0.2±1.0	−0.7±0.6	0.2±0.1	$0.0\pm 4.9\times 10^{-3}$	$0.0\pm 6.2\times 10^{-3}$	$0.0\pm 1.0\times 10^{-2}$	$-2.0\pm 9.8\times 10^{-2}$	$0.0\pm 2.5\times 10^{-2}$	$-0.1\pm 3.6\times 10^{-2}$

For both scenarios, the average errors in parameters describing the virtual source point were less than or equal to 0.7 mm. The average errors observed in parameters describing rotations of the source and detector arrays were less than 0.01 deg. The piercing point coordinates ($u_{o}$, $v_{o}$) were estimated with average errors less than or equal to 0.1 mm for both cases considered. Errors in the ($u_{o}$, $v_{o}$) coordinates were less than the dimension of a virtual detector element. The maximum error observed in the SDD parameter estimation was −2.0  mm. Potential techniques to improve estimates of the SDD parameter are discussed in Sec. 4 of this paper.

### 3.3 Experimental Validation

The estimated geometric parameters for the bench-top SBDX setup describing SDD, piercing point $\left(u_{o},v_{o}\right)$, and rotation $\left(\theta_{x},\theta_{y}\right)$ are presented in Table 6, averaged across the view angle. The mean estimated SDD was 1500.0 mm, compared to a nominal value of 1500.0 mm. The proposed calibration method estimated the source and detector rotation about the x-axis to be 0.2 deg and rotation about the y-axis to be 0.5 deg.

**Table 6:** Estimated geometric parameters (SDD, $\theta_{x}$, $\theta_{y}$, $u_{o}$, $v_{o}$) for the bench-top SBDX setup.

Parameter	Value
SDD (mm)	1500.0±0.1
$\theta_{x}$ (deg)	0.16±0.03
$\theta_{y}$ (deg)	0.46±0.03
$u_{o}$	355.5±0.0
$v_{o}$	355.5±0.0

**Fig. 5:**
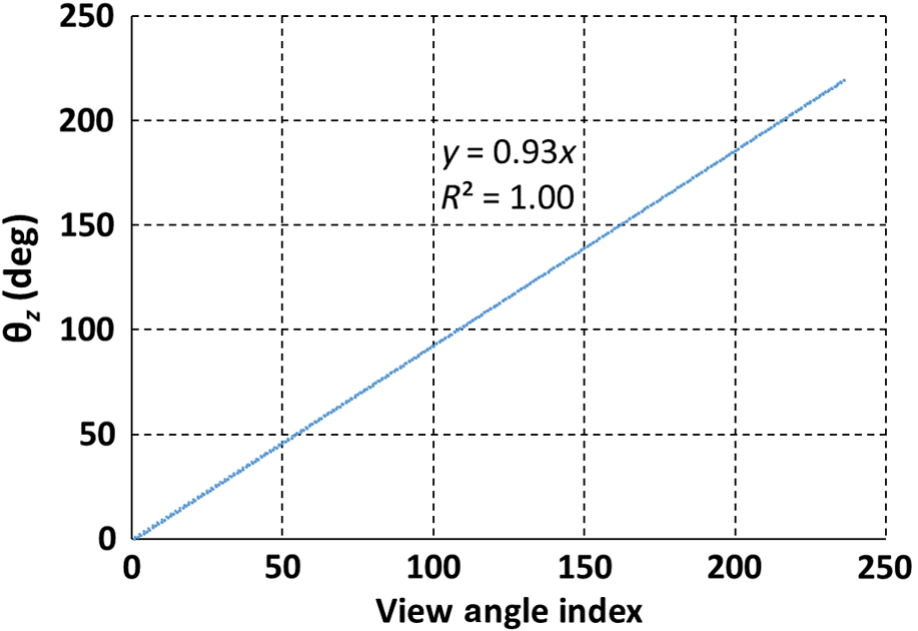
The estimated view angle ($\theta_{z}$) is plotted versus the view angle index.

The estimated view angle $\theta_{z}$ is plotted versus the view index in Fig. 5. A linear regression was performed to determine the relationship between view index and view angle. The slope of the linear regression line was 0.93 deg per view index and the intercept was 0.0 deg. The angular increment between view indices determined from the slope of the regression line was in good agreement with the motion controller programming of 0.93±0.05  deg per view index.

#### 3.3.1 Atrium phantom results

**Fig. 6:**
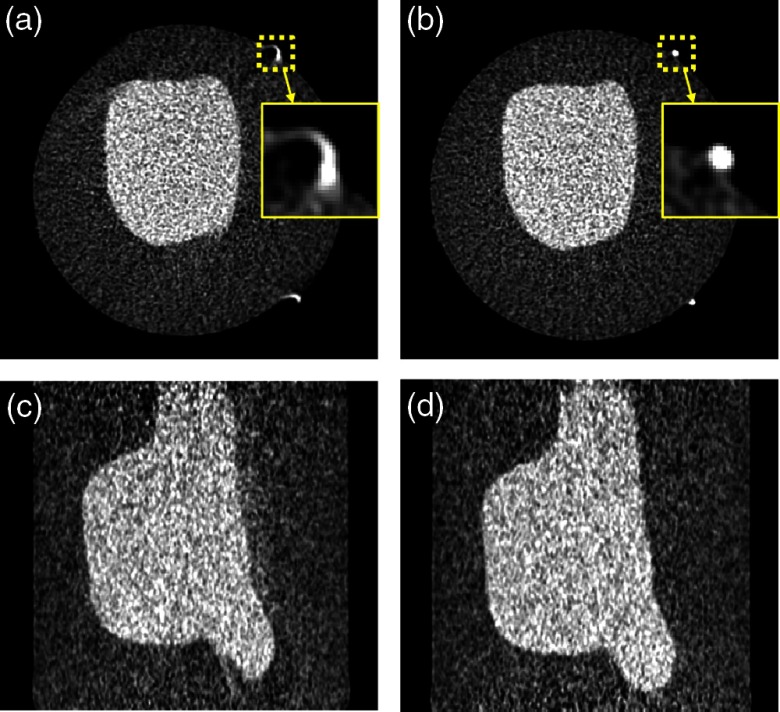
SBDX projection data reconstructed without geometric calibration: (a) shows double contour artifacts and blurring of object structures. (b) The artifacts are eliminated when geometric calibration is used during image reconstruction. Sagittal slices without (c) and with (d) geometric calibration are presented. Display window is [−300,700] HU.

The exact geometric parameters of the system (e.g., the exact distance between the tungsten target and the surface of the CdTe detector) were not measured directly due to the invasive and destructive nature of such measurements. However, the performance of the proposed method can be assessed qualitatively by comparing reconstructed CT images without [Figs. 6(a) and 6(c)] and with geometric calibration [Figs. 6(b) and 6(d)]. Double contour artifacts are observed near the high-contrast fiducials and object blurring is present for the image reconstructed without geometric calibration. The artifacts are reduced in the image reconstructed with geometric calibration.

#### 3.3.2 Spatial fidelity results

**Fig. 7:**
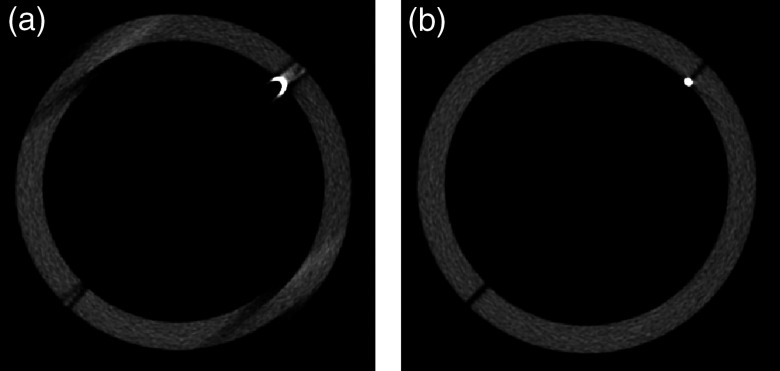
IGCT reconstruction without geometric calibration: (a) shows artifacts. (b) Contour artifacts around a steel fiducial are reduced in an image reconstructed with calibration. Display window is [−600,1000] HU.

**Fig. 8:**
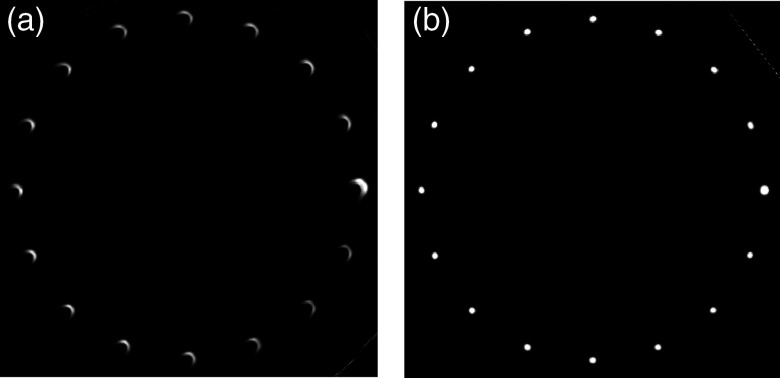
(a) MIP image demonstrates contour artifacts around high-contrast steel fiducials for an image reconstructed without geometric calibration. (b) Application of the proposed calibration technique reduced the artifacts.

The reconstructed helix phantom is shown in Fig. 7(a) without geometric calibration and in Fig. 7(b) with geometric calibration. The PMMA cylinder supporting the helix is blurred in the image without calibration and artifacts are present near the steel fiducial. A maximum intensity projection (MIP) image shows contour artifacts for each of the steel fiducials in the image reconstructed without calibration [Fig. 8(a)]. Geometric calibration reduced the artifacts [Fig. 8(b)].

Spatial fidelity was determined by comparing IGCT-derived fiducial separation distances to the reference distances computed from the known helix geometry. In the image reconstructed without geometric calibration, the mean error in fiducial separation distance was 0.58 mm and the standard deviation was 5.90 mm. Application of geometric calibration during reconstruction reduced the mean error to −0.04  mm and the standard deviation to 0.18 mm, indicating improved spatial fidelity.

#### 3.3.3 Modulation transfer function

**Fig. 9:**
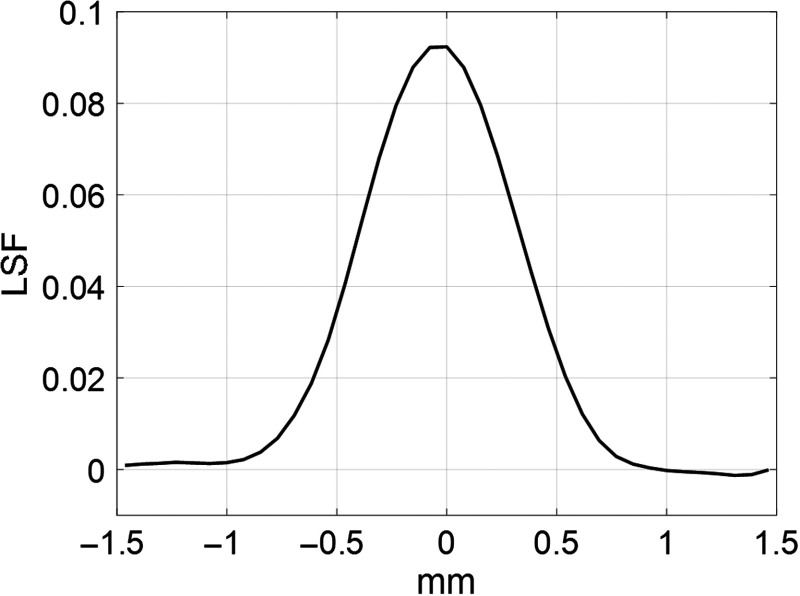
LSF derived from imaging of a 0.154-mm-diameter steel wire. The FWHM value measures 0.81 mm.

**Fig. 10:**
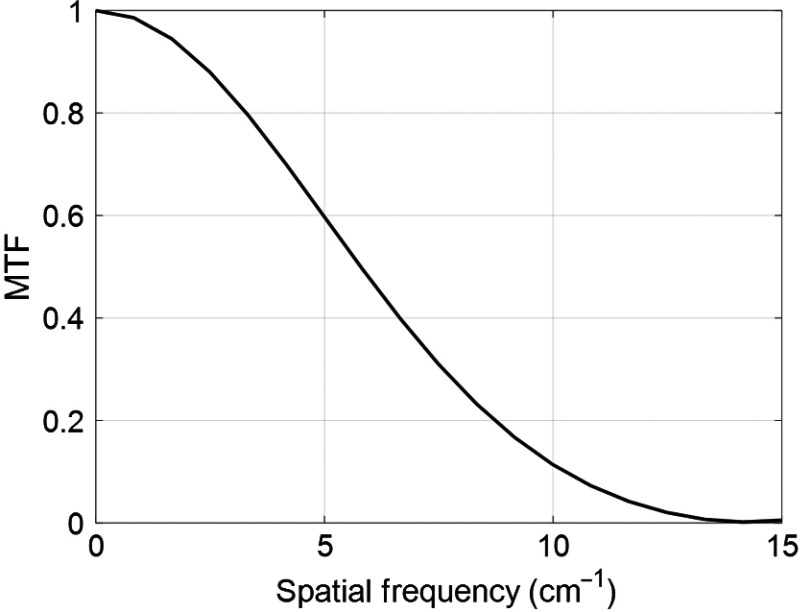
The MTF using IGCT geometric calibration is shown. The 10% MTF value corresponds to a spatial frequency of $10.3\;{\mathrm{cm}}^{-1}$.

Figure 9 shows the LSF derived from an SBDX CT image of a wire phantom using the proposed geometric calibration method. The full width at half maximum (FWHM) of the LSF measured 0.81 mm. For context, we note that SBDX CT is being investigated for its potential to provide 3-D anatomic maps during catheter-based interventions. The measured FWHM value (0.81 mm) of the LSF is less than the diameter (2 to 3 mm) of a typical ablation catheter tip that would be displayed with the 3-D anatomic map.^15^ Figure 10 presents the MTF curve. The MTF value was 50% at a spatial frequency of $5.8\;{\mathrm{cm}}^{-1}$ and 10% at $10.3\;{\mathrm{cm}}^{-1}$. The LSF and MTF are presented as example resolution metrics in the presence of geometric calibration. No attempt was made to optimize reconstruction parameters to maximize spatial resolution.

## 4 Discussion

This paper presents a single-view geometric calibration method for IGCT. Single-view calibration is an important step toward enabling IGCT on flexion-prone C-arm systems. The calibration method presented in this paper was inspired by the P-matrix approach used in conventional CT.^12^ The SBDX composite image is analogous to a virtual cone-beam projection originating at the center of the detector array. This was exploited to estimate geometric parameters by parameterizing the SBDX system properties used to construct the projection matrix. A limitation of this approach is that the 2-D detector array is reduced to a single point referred to as the virtual source point. As a result, the proposed calibration technique assumes that rotations or translations of detector and source arrays occur in unison. Nonetheless, experimental data demonstrated the proposed method’s ability to reduce artifacts caused by geometric uncertainties. Future work will investigate extending the P-matrix approach presented here to use a stereoscopic imaging method to estimate rotations and translations of the source and detector arrays independent of one another.^6^

The calibration method was evaluated through numerical simulations and experimentally acquired projection data. For both scenarios, the proposed method reduced the image artifacts that were observed in reconstructions performed without calibration. The rRMSE was reduced from 7.7% to 0.4% for the reconstruction of a numerical thorax phantom. The sensitivity of the method to uncertainties in SDD, translations, and rotations was examined through numerical simulations. The sensitivity analysis showed that uncertainties in parameters describing translation or rotation could be estimated with average errors on the order of 0.7 mm and 0.01 deg. The most challenging parameter to estimate accurately was the SDD. The proposed method considered only information contained in the single multiplane composite image during calibration. Future work could investigate using the 3-D information contained in the tomosynthetic plane stack^4^ to reduce errors observed in the SDD parameter estimation.

A potential alternative to the technique examined here is to determine a P-matrix for each individual source and detector pair using raw IGCT projection data. Although this would provide a more complete characterization of the imaging geometry, accurate localization of fiducials in the many individual low-flux detector images is an expected challenge. An advantage of the approach pursued here is that fiducials can be easily detected and localized in the “virtual projection” composite image that is formed following SAA tomosynthesis reconstruction.

The initial experimental work performed here used a rotating phantom stage to mimic C-arm rotation with precisely known rotation increments. However, the SBDX source and detector are mounted to a C-arm with rotational capability. Future work will include applying the new calibration procedure to projection data acquired with a C-arm rotation of the SBDX source and detector arrays.

## 5 Conclusions

A method for single-view geometric calibration of a C-arm IGCT system was demonstrated. The proposed calibration method was shown to suppress or remove image artifacts due to uncertainties in the imaging system geometry through simulations and a bench-top setup with a rotating stage. Future work will apply and evaluate the proposed method using phantom data acquired in SBDX C-arm rotational scans. The development of geometric calibration techniques is an important step toward developing C-arm inverse geometry computed tomography for SBDX.
